# Hydrolysis of *Agave fourcroydes* Lemaire (henequen) leaf juice and fermentation with *Kluyveromyces marxianus* for ethanol production

**DOI:** 10.1186/1472-6750-14-14

**Published:** 2014-02-14

**Authors:** Pablo A Villegas-Silva, Tanit Toledano-Thompson, Blondy B Canto-Canché, Alfonso Larqué-Saavedra, Luis F Barahona-Pérez

**Affiliations:** 1 Centro de Investigación Científica de Yucatán AC, Calle 43 No. 130 Col. Chuburná de Hidalgo, Mérida, Yucatán 97200, Mexico

**Keywords:** Biofuel, Sugars, Oligofructans, Hydrolysis, Pretreatments

## Abstract

**Background:**

Carbon sources for biofuel production are wide-ranging and their availability depends on the climate and soil conditions of the land where the production chain is located. Henequen (*Agave fourcroydes* Lem.) is cultivated in Yucatán, Mexico to produce natural fibers from the leaves, and a juice containing fructans is produced during this process. Fructans can be hydrolyzed to fructose and glucose and metabolized into ethanol by appropriate yeasts. In Mexico, different *Agave* species provide the carbon source for (distilled and non-distilled) alcoholic beverage production using the stem of the plant, whilst the leaves are discarded. In this work, we investigated the effect of thermal acid and enzymatic hydrolysis of the juice on the amount of reducing sugars released. Growth curves were generated with the yeasts *Saccharomyces cerevisiae* and *Kluyveromyces marxianus* and fermentations were then carried out with *Kluyveromyces marxianus* to determine alcohol yields.

**Results:**

With thermal acid hydrolysis, the greatest increase in reducing sugars (82.6%) was obtained using 5% H_2_SO_4_ at 100°C with a 30 min reaction time. Statistically similar results can be obtained using the same acid concentration at a lower temperature and with a shorter reaction time (60°C, 15 min), or by using 1% H_2_SO_4_ at 100°C with a 30 min reaction time. In the case of enzymatic hydrolysis, the use of 5.75, 11.47 and 22.82 U of enzyme did not produce significant differences in the increase in reducing sugars. Although both hydrolysis processes obtained similar results, the difference was observed after fermentation. Ethanol yields were 50.3 ± 4 and 80.04 ± 5.29% of the theoretical yield respectively.

**Conclusions:**

Final reducing sugars concentrations obtained with both thermal acid and enzymatic hydrolysis were similar. *Saccharomyces cerevisiae*, a good ethanol producer, did not grow in the hydrolysates. Only *Kluyveromyces marxianus* was able to grow in them, giving a higher ethanol yield with the enzymatic hydrolysate. The leaves account for a non-negligible weight of the total agave plant biomass, so this work complements the knowledge already developed on agave fermentations by making it possible to produce ethanol from almost the entire plant (stem and leaves).

## Background

The use of biofuels such as bioethanol, biodiesel and biogas in the transport sector offers a valuable alternative in order to minimize greenhouse gas emissions and fossil-fuel dependence, and is being developed in many countries. Bioethanol is currently produced at competitive prices from two main raw materials: sugar and starch [1]. However, in order to avoid the conflict between using land for food or energy production, the use of forestry and/or agricultural residues for bioethanol production is attracting the interest of scientists with a view to establishing economical and sustainable processes. The availability of local raw materials is therefore an important issue that has to be addressed when attempting to assess their viability. Carbon sources for bioethanol production are wide-ranging [2] and their availability depends on the climate and soil conditions of the land where the production chain is located. The north of the Yucatán peninsula, Mexico, consists mainly of semi-arid lands with poor soils that do not support the cultivation of sugar cane. Corn is cultivated on a small scale, but cannot be used for fuel purposes because this crop forms the basis of the traditional Mexican diet. Nevertheless, agaves can grow well in these soils due to the fact that their metabolism is adapted to arid conditions [3]. Although biomass productivity of henequen (*Agave fourcroydes* Lem.) is not as high as sugar cane or sweet sorghum, it is similar to a variety of other energy crops, as shown in Table 1[4,5]. Henequen is cultivated in this region to produce natural fibers from the leaves, and a juice containing fructans is produced during this process [6]. These fructans can be hydrolyzed to fructose and glucose and metabolized into ethanol by appropriate yeasts [7]. In Mexico, different *Agave* species provide the carbon source for (distilled and non-distilled) alcoholic beverage production and both traditional and modern processes have been discussed at length in the literature [8]. They all use the stem of the plant as the source of sugars, whilst the leaves are discarded. The stems are cooked to hydrolyze the fructan chains into their monomers, and pressed to extract a fermentable sugar-rich juice. In the case of henequen, the older leaves at the base of the plant are harvested every 3-4 months and decorticated to obtain the fibers. During processing, water is used to wash the fibers, resulting in the dilution of the components present in the juice.

**Table 1:** **Comparison of *Agave fourcroydes *** **L. biomass productivity with other energy crops**

**Crop**	**Dry biomass productivity (t∙ha**^**-1**^**∙year**^**-1**^**)**
*Agave fourcroydes*	15
*Agave tequilana*	25
Sugarcane	50-67
Corn	17-18
Sugar beet	11-17
Sweet sorghum	24-47
Switchgrass	10-15
Miscanthus	10-13

Although producing ethanol from the juice (stem and/or leaves) of agaves is not a direct fermentation of simple sugars (as with sugar cane or sweet sorghum), it is not as difficult as producing it from lignocellulosic materials. There is no need for solid material pretreatments, but a hydrolysis step is needed because fermentable sugars are not directly available. Unlike fructans from chicory, which are mainly composed of fructose molecules joined by β(2-1) linkages, agave fructans contain an important amount of β(2-6) linkages that result in branched molecules [7,9,10]. This can affect, for example, the choice of enzyme for the hydrolysis of the fructan chains. Commercial enzyme preparations that contain endo- and exo-inulinases can be used to achieve good yields in agave fructans hydrolysis. Inulinases can mainly be produced by microorganisms such as yeast strains (*Candida* sp., *Sporotrichum* sp., *Pichia* sp., and *Kluyveromyces* sp.) or fungi (*Aspergillus* and *Penicillium* species) [11]. Thermal acid hydrolysis can also be used, but this process is associated with the production of unwanted by-products (hydroxymethyl furfural and fructose dianhydride) that can affect further biological steps or the purity of final products [12].

The juice from the leaves of the henequen plant has previously been studied for ethanol production [6]. In that work, the juice was diluted to simulate the concentrations obtained from the mills. The low sugar content was compensated for by the addition of molasses, another industrial residue. However, technical proposals exist to obtain pure juice from the mills, meaning that sugar concentrations could be higher and there would be no need to add other carbon sources. The use of a mixture of yeast strains, *Saccharomyces cerevisiae* and *Kluyveromyces marxianus*, helped to improve ethanol yields, although they were still low. The aim of this work was to investigate the effect of thermal acid and enzymatic hydrolysis of the juice on the amount of reducing sugars released from these processes. Temperature, time and sulfuric acid concentrations were varied for thermal acid hydrolysis. Temperature and enzyme concentration were varied for enzymatic hydrolysis. The treatment that gave the highest reducing sugars yields was used to continue the study. Growth curves were generated with the yeasts *Saccharomyces cerevisiae* and *Kluyveromyces marxianus* and fermentations were then carried out with *Kluyveromyces marxianus* to determine alcohol yields.

## Results and discussion

### Thermal acid hydrolysis

**Figure 1:**
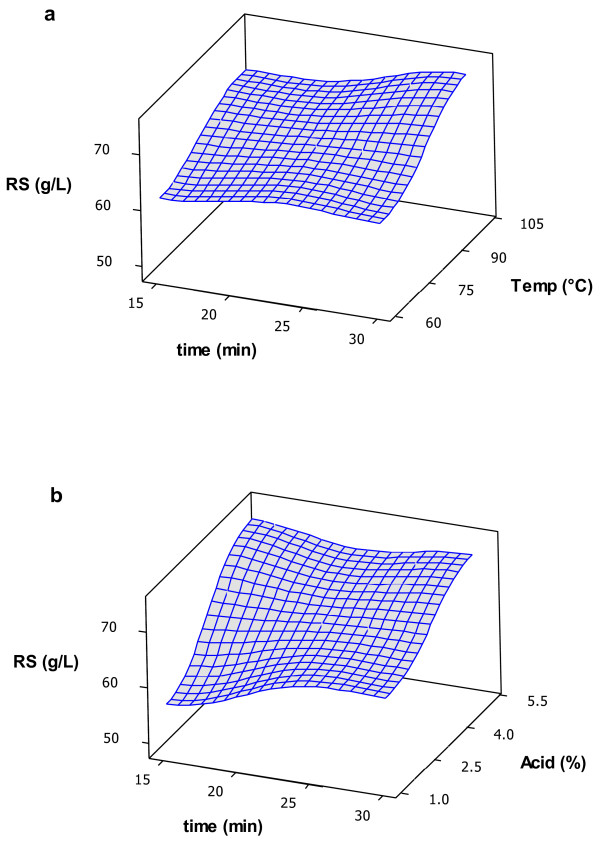
**Response surfaces for the effect of a) reaction temperature and b) H**_**2**_**SO**_**4 **_**concentration on reducing sugars concentration.**

**Figure 2:**
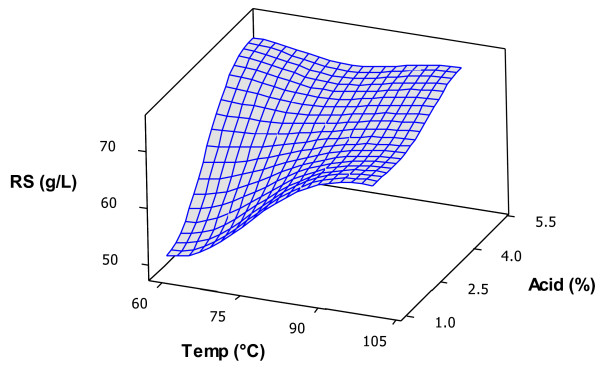
**Response surface for the combined effect of reaction temperature and H**_**2**_**SO**_**4 **_**concentration on reducing sugars concentration.**

The juice obtained from henequen leaves had a soluble solids concentration of 10°Bx, which was similar to that obtained previously [6], and an initial reducing sugars concentration of 40.2 ± 0.64 g∙L^-1^. Time had little effect on thermal acid hydrolysis of the juice, as can be seen in the surface response graphs in Figure 1. The reducing sugars concentrations remained almost the same when reaction temperature (Figure 1a) or acid concentration (Figure 1b) were kept the same and only time was changed. Acid concentration showed a greater effect on reducing sugars release than reaction temperature within the ranges employed in this work. As expected, a lower release of reducing sugars was obtained when lower values were used for acid concentration and reaction temperature, as shown in Figure 2. Good yields were also obtained at low acid concentrations and high temperatures. Reducing sugars values after the thermo-acid treatments are shown in Table 2. The greatest increases in reducing sugars concentrations were obtained when 5% H_2_SO_4_ was added to the juice. The only experiment that produced a statistically similar increase in reducing sugars using 1% H_2_SO_4_ was treatment 7, carried out at 100°C for 30 min. Although it requires more energy and a longer reaction time, the amount of H_2_SO_4_ is 5 times lower and neutralization requires less NaOH. Treatment 7 conditions were used in further experiments in this work. The selection of parameters and their ranges depends not only on better results, but also on economic, environmental and practical factors [13]. For larger-scale production, procedures requiring smaller energy inputs are preferred, and even Treatment 4 conditions could be used without a detrimental effect on reducing sugars production. To what extent the use of less H_2_SO_4_ or lower temperature and reaction times produce greater savings is beyond the scope of this work, but should be studied. There are few reports on agave fructan hydrolysis, and most of them are on the stems of *Agave tequilana* W. because this is used for tequila production [9,10]. Waleckx et al. recently reported that 98% hydrolysis efficiency was obtained after 25.5 h of stem cooking [7]. However, it was possible to use shorter heating times in this work due to the liquid nature of the juice. Avila et al. [14] reported a hydrolysis efficiency of greater than 90% at 60°C with a 10 minute reaction time after fructans from *A. tequilana* stems were extracted and then thermally hydrolyzed using 0.54 N HCl.

**Table 2:** Increase in reducing sugars after thermal acid hydrolysis of henequen juice

**Treatment**	**Tempeature (°C)**	**Heating time (min)**	**H**_**2**_**SO**_**4 **_**conc. (%v/v)**	**Reducing sugars**^**2 **^**(g L**^**-1**^**)**	**Reducing sugars increase (%)**
Reference^1^	--	--	--	40.25 ± 0.64^a^	--
1	60	15	1	48.84 ± 2.58^b^	21.3
2	60	15	5	74.59 ± 1.05^c^	85.3
3	60	30	1	53.55 ± 1.83^d^	33
4	60	30	5	73.36 ± 1.7^c^	82.3
5	100	15	1	64.38 ± 2.3^e^	60
6	100	15	5	74.01 ± 1.49^c^	83.9
7	100	30	1	74.4 ± 3.29^c^	84.8
8	100	30	5	74.95 ± 1.41^c^	86.2

^1^Raw henequen leaf juice was used as a reference.

^2^Different letters indicate statistically significant differences.

### Enzymatic hydrolysis

**Figure 3:**
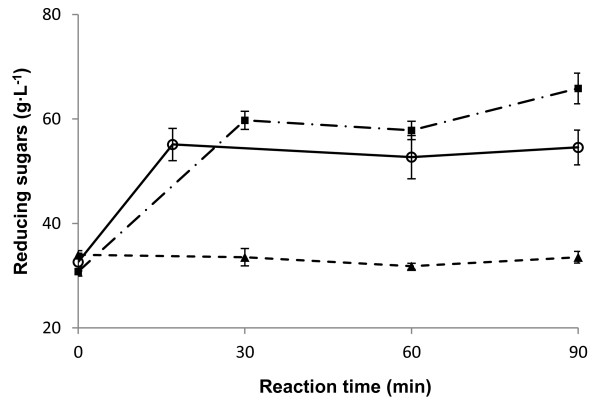
**Effect of temperature on enzymatic hydrolysis of henequen juice.** (■) 60°C; (○) 50°C; (▲) without enzyme at both 50°C and 60°C.

The effect of temperature on reducing sugars concentration during enzymatic hydrolysis of henequen juice is shown in Figure 3. As can be seen, the maximum release of reducing sugars is obtained after a 30 min reaction at both temperatures (50 and 60°C) using 22.82 U of enzyme as indicated by the manufacturer. A slightly higher concentration of reducing sugars was obtained at 60°C. The reference (henequen juice without enzyme) was also conducted at 50 and 60°C and the results were the same for both temperatures, showing that no increase in reducing sugars concentrations occurred without enzyme. It has been reported [11] that inulinase from the marine yeast *Pichia guilliermondii* is most active at 60°C. However, Ricca et al. [15] reported that higher inulinase activity is obtained at 60°C, but 30% activity loss occurred after 5 h. This must be considered when scaling-up the process, as procedures are longer than at laboratory level, meaning that lower temperatures may give better results. In order to obtain the maximum concentration of reducing sugars, experiments on enzymatic hydrolysis of henequen juice were conducted at 60°C in this study.

**Figure 4:**
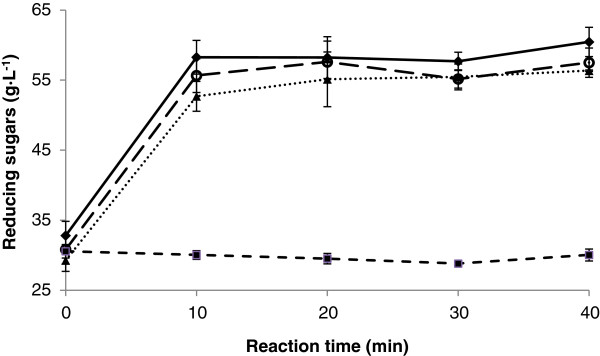
**Effect of enzyme concentration on enzymatic hydrolysis of henequen juice.** (♦) 22.82 U; (○) 11.47 U; (▲) 5.75 U; (■) without enzyme.

To determine the effect of enzyme concentration on reducing sugars release, lower concentrations than 22.82 U were tested. As can be seen in Figure 4, hydrolysis of the fructans present in the juice occurs during the first 10 min of the reaction. This experiment also showed that the use of 4 times less enzyme (5.75 U) is enough to attain the same levels of reducing sugars release as using 22.82 U. This suggests that there is no saturation of the enzyme at lower levels. There were no significant differences in the increase in reducing sugars using 5.75, 11.47 and 22.82 U of enzyme. The reference was henequen juice incubated under the same conditions without enzyme; in this case, no change in reducing sugars concentrations was observed. The concentrations of reducing sugars obtained by enzymatic hydrolysis are similar to those obtained by thermal acid hydrolysis.

### Fermentation

**Figure 5:**
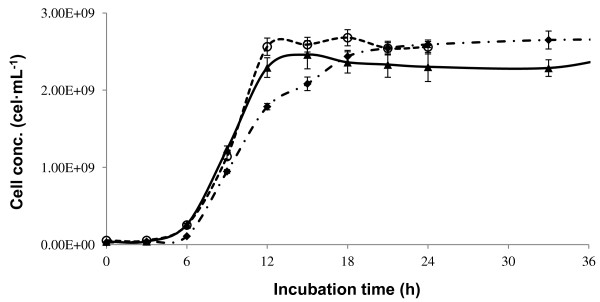
**Growth curves of *Kluyveromyces marxianus*.** Enzymatic (▲), thermal acid (♦) and non-hydrolyzed (○) henequen juice hydrolysates.

A growth curve was first generated for the yeast *Saccharomyces cerevisiae* with both thermo-acid and enzymatic henequen juice hydrolysates. The yeast did not grow in either of the hydrolysates. This behavior could be due to the presence of inhibitory compounds in the henequen juice. It is well documented that agave plants produce saponins with inhibitory activity [16,17]. To corroborate this, Cira et al. [18] produced a modified strain of *S. cerevisiae* which resists the saponins present in the musts of *Agave tequilana* Weber var. azul and *Agave salmiana*, and succeeded in fermenting them into ethanol. Saponins can be hydrolyzed to their corresponding sapogenins, but higher acid concentrations and longer reaction times than the ones used in this work are needed [19]. This lack of performance has been reported in the literature [20], where lower ethanol yields were obtained with *Saccaromyces cerevisiae* (70-76%) compared to a *Kluyveromyces marxianus* strain (92-96%) when fermenting *Agave tequilana* musts. On the contrary, the yeast *Kluyveromyces marxianus* was able to grow in the henequen juice hydrolysates, as shown in Figure 5. The yeast could also grow in raw juice (the juice was only heated to 80°C and cooled immediately to lower microbial contamination). This can be explained by the fact that the strain used in this work was isolated from the henequen plant and is adapted to the metabolites present in the juice or has the enzymes to hydrolyze them. In a spontaneous fermentation of *A. fourcroydes* stem must, a great diversity of yeasts was present and *K. marxianus* and *S. cerevisiae* were the dominant species by the end of the fermentation [Dr. Patricia Lappe, personal communications]. *K. marxianus* presented a lag phase of approximately 6 hours. In Figure 5, it can be seen that the yeast performed better in the enzymatic hydrolysate and in the raw juice than in the thermal acid hydrolysate at the beginning of the growth curves. It has been reported that materials subjected to acid hydrolysis are harder to ferment [21]. After 18 hours of growth, cell concentrations were the same in both hydrolysates.

**Figure 6:**
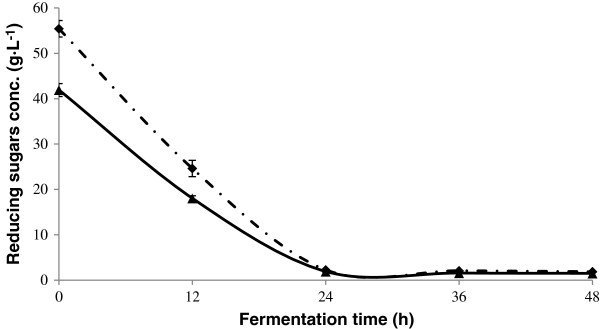
**Reducing sugars consumption during fermentation of henequen juice hydrolysates.** Enzymatic (▲) and thermal acid (♦) hydrolysates.

**Figure 7:**
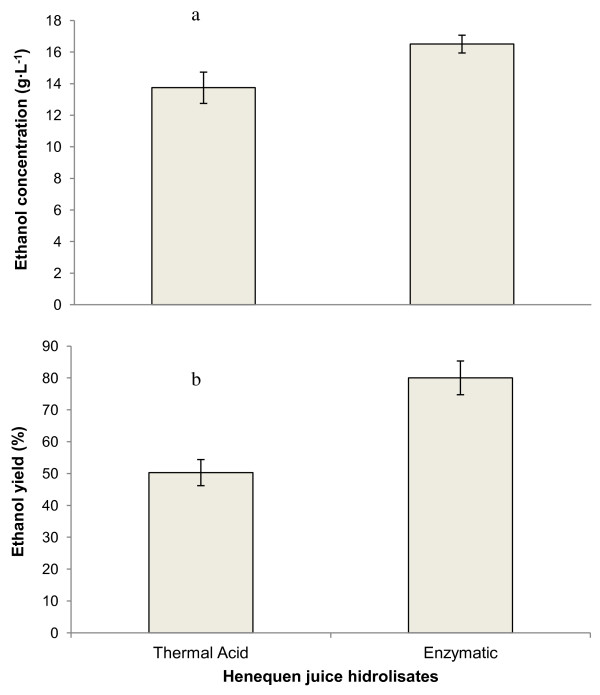
Comparison of a) ethanol concentrations and b) ethanol yields obtained after fermentation of thermal acid and enzymatic hydrolysates of henequen juice.

Alcoholic fermentations were carried out with enzyme and thermal acid hydrolyzed henequen juice. Reducing sugars were consumed during the first 24 hours of fermentation as shown in Figure 6. After 48 hours of fermentation, ethanol concentrations were 13.74 ± 0.99 g · L^-1^ for the thermal acid hydrolyzed juice and 16.5 ± 0.56 g · L^-1^ for the enzyme-hydrolyzed juice. As shown in Figure 7a, the difference between the two results was found to be statistically significant. Based on the amount of reducing sugars consumed during the fermentations, ethanol yields of 50.3 ± 4 and 80.04 ± 5.29% were obtained respectively. As show in Figure 7b, the difference between the two results was statistically significant. The yeast was able to consume the reducing sugars, but did not efficiently transform them into ethanol in the case of the thermal acid hydrolysate. Ethanol yields depend on fermentation conditions. When inulinase-hydrolyzed artichoke tubers were fermented using a strain of *S. cerevisiae*, yields of 80-84% of the theoretical value were obtained [22] which are similar to those obtained in this work. This indicates that *S. cerevisiae* can assimilate the sugars released by an enzymatic hydrolysis of oligofructans, so henequen leaves contain compounds that inhibit growth of this yeast. Ethanol yields of up to 90% of the theoretical value were obtained when *S. cerevisiae* was used to ferment YM broth with nitrogen supplementation [23]. In another study carried out in our laboratories, the same *S. cerevisiae* strain used in this work gave an ethanol yield of 98% of the theoretical value when a thermal acid hydrolysate of jatropha kernel fruit was fermented. The yeast *S. cerevisiae* is known for its higher tolerance to ethanol concentrations [24], whilst *K. marxianus* is known for its thermotolerance [25,26].

The selection of one yeast over the other depends on the nature of the must and fermentation conditions. The use of a mixture of both yeasts can even be envisaged [6].

**Figure 8:**
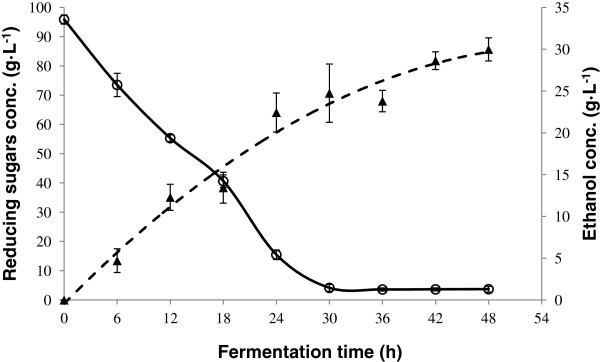
Reducing sugars consumption (○) and ethanol production (▲) during fermentation of an enzymatic hydrolysate of henequen juice.

An ethanol production curve was generated using an enzymatic hydrolysate. This juice was obtained during the dry season and was more concentrated. It has been documented that the juice of henequen stems is more concentrated in the dry season [27] and we found the same effect with the leaf juice. It was decided not to dilute the juice to find out if there was any effect on the hydrolysis and fermentation processes. A higher concentration of reducing sugars was obtained after enzymatic hydrolysis, indicating that the amount of enzyme chosen previously can perform well with any juice concentration obtained during the year. Reducing sugars uptake and ethanol production are shown in Figure 8. It can be seen that after 30 hours of fermentation, sugar consumption is already complete with no statistically significant differences in ethanol concentrations until 48 h. An ethanol yield of 77 ± 5.86% of the theoretical yield was obtained.

With the higher yields obtained in this work, it is possible to produce 0.038 L of ethanol per liter of leaf juice. Taking into account that a henequen leaf contains 0.78-0.89 L of juice and approximately 250 million leaves are processed per year, 7.125 million liters of ethanol could therefore be produced with this residue. As for production costs, they will be higher than for processes employing direct fermentation of simple sugars, such as sugar cane or sweet sorghum juice, due to the inclusion of the enzymatic hydrolysis step. The amount of enzyme used, however, is minimal. Production costs will be lower than for solid lignocellulosic residues, because there is no pre-treatment step before saccharification. It is easier and cheaper to process liquid materials.

To our knowledge, no data has previously been reported on hydrolyzed agave leaf juice fermentations to produce ethanol.

## Conclusions

Final reducing sugars concentrations obtained with both thermal acid and enzymatic hydrolysis were similar, but enzymatic hydrolysis gave better ethanol yields after fermentation. *Saccharomyces cerevisiae*, a good ethanol producer and one used in agave stem must fermentations, could not grow in either hydrolysate. The development of strains resistant to inhibitory compounds present in agave leaves is important for commercial exploitation of this part of the plant. Fermentation of thermal acid and enzymatic hydrolysates with *Kluyveromyces marxianus* resulted in theoretical ethanol yields of 50.3 ± 4 and 80.04 ± 5.29% respectively. This yeast is capable of producing inulinase, but attempts to ferment the leaf juice without hydrolysis failed, meaning that preliminary hydrolysis of the fructans present in the juice appears to be necessary. *K. marxianus* can also be manipulated in order to attain higher ethanol yields. These studies, together with the isolation of new strains from henequen leaf juice, are being carried out by our group. This work complements the knowledge already developed on agave juice fermentation. Agave leaves (*fourcroydes* or *tequilana*) account for a non-negligible weight of total plant biomass and comprise an important carbohydrate source that can be used to produce ethanol as biofuel.

## Methods

### Reagents

All reagents were analytical grade. Inulinase enzyme (Sigma-Aldrich) was obtained from *Aspergillus niger* with a declared activity of 2000 U · mL^-1^ and a density of 1.13 g · cm^-3^. One enzymatic unit is defined as the amount of enzyme necessary to release one g of reducing sugars per minute at 50°C.

### Plant material

Henequen leaves were obtained from healthy plants grown in the gardens of Centro de Investigación Científica de Yucatán AC. They were rinsed with tap water and passed through a three-roller mill to extract the juice, which was filtered to eliminate solid leaf residues and stored at -20°C until use.

### Thermal acid hydrolysis

The variables considered important in this process were temperature, heating time and H_2_SO_4_ concentration. A 3^2^ full factorial design leading to eight sets of experiments was used to determine the effect of the above mentioned variables on the release of reducing sugars. The levels for each variable were determined on the basis of previous experience with henequen juice hydrolysis and were decided as follows: temperature = 60 and 100°C, heating time = 15 and 30 min, H_2_SO_4_ concentration = 1 and 5% (v/v). These levels were decided not only based on reaction system characteristics, but also based on economic and practical factors. Thermal acid hydrolysis experiments were carried out in 250 mL Erlenmeyer flasks with 80 mL of henequen leaf juice. Fresh juice was used as a reference for the initial reducing sugars concentration.

### Enzymatic hydrolysis

To study enzymatic hydrolysis, a more classical (one variable at a time) approach was used due to the lack of previous data. Conditions of preliminary assays were based on enzyme data provided by the manufacturer. They were carried out in 25 mL Erlenmeyer flasks with 5 mL of raw henequen juice at pH 4.5. Two temperatures, 55 and 60°C, were tested using 22.82 U of enzyme and 90 min incubation time. Subsequently, three enzyme concentrations were tested at 60°C: 5.75, 11.47 and 22.82 U of enzyme and 40 min incubation time.

For fermentation assays, enzymatic hydrolysis was carried out in 250 mL Erlenmeyer flasks with 180 mL of raw henequen juice. An enzyme concentration of 5.75 U was used. Incubation was carried out at 60°C for 30 min and pH 4.5. All enzymatic hydrolysis incubations were performed at 150 rpm.

### Yeast strains

*Kluyveromyces marxianus* was isolated from the base of henequen leaves following classical isolation procedures. It was characterized by phenotypical and molecular tests [28]. Commercial *Saccharomyces cerevisiae* was obtained from Safmex S.A. de C.V. (Mexico). Both yeasts were maintained in Petri dishes on YPGA medium containing glucose (20 g∙L^-1^), yeast extract (5 g∙L^-1^), peptone (10 g∙L^-1^) and agar (20 g∙L^-1^), and incubated at 30 ± 2°C in darkness. The cultures were collected in sterile water and kept at 4°C until use. Viability of the cell suspensions was measured by staining with methylene blue. Cell suspension viabilities ≥ 95% of both strains were used in all experiments.

### Growth curves

Growth curves were generated in 250 mL Erlenmeyer flasks with 100 mL of thermal acid or enzyme hydrolyzed henequen juice adjusted to pH 4.5. Prior to hydrolysis, soluble solids of the raw henequen juice were adjusted to 6°Bx. Ammonium sulfate (1.5 g∙L^-1^) was used as the nitrogen source [23] and a cell concentration of 3×10^7^ cell∙mL^-1^ was employed in all cases. The flasks were incubated at 30 ± 2°C with 150 rpm agitation for 33 h. Samples were harvested every 3 h. Non-hydrolyzed henequen juice was used as a reference. In this case, to avoid contamination, the juice was heated to 80°C and immediately cooled to room temperature after adjusting soluble solids to 6°Bx.

### Fermentations

Inocula for fermentations were prepared with thermal acid or enzymatically hydrolyzed henequen juice with a soluble solids concentration of 6°Bx and an initial cell concentration of 3×10^7^ cell∙mL^-1^. pH was adjusted to 4.5 and 1.5 g∙L^-1^ ammonium sulfate was added. Growth was maintained at 30 ± 2°C for 18 hours. The amount of the inoculum was 10% of total fermentation volume. Fermentations were carried out in 250 mL Erlenmeyer flasks with 180 mL of non-diluted thermal acid or enzymatically hydrolyzed henequen juice. pH was adjusted to 4.5 and 1.5 g∙L^-1^ ammonium sulfate was added. After inoculation, the flasks were kept at 30 ± 2°C for 48 hours without agitation.

### Analytical methods

Soluble solids concentration was measured with a portable refractometer (Cole-Parmer FG103/113) and expressed as °Bx. Reducing sugars concentrations were determined in the hydrolysates and during fermentations by the 3, 5-dinitrosalicilic acid method [29]. The absorbance of the samples was read at 550 nm. For ethanol quantification, 25 mL of fermented sample was diluted with 25 mL of distilled water and distilled at 100°C until 25 mL of distillate was recovered. Ethanol concentration was determined by the dichromate method [30]. The absorbance of the samples was read at 585 nm.

### Statistical analysis

All experiments were performed in triplicate (n = 3). For statistical analysis, the Statgraphics Centurion XV (Statpoint technologies Inc., Warrenton, VA, USA) software was employed. Significant differences among treatments were determined using the Tukey HSD test with p ≤ 0.05. For response surface analysis, the MINITAB 16 (Minitab Inc., State College, PA, USA) software was used.

## Competing interests

The authors declare that they have no competing interests.

## Authors’ contributions

PAVS carried out the experimental work and participated in the design of the study. TTT participated in the reducing sugar and statistical analysis. BBCC participated in the design and analysis of the enzymatic hydrolysis experiments. ALS participated in the design of the study and commented on the manuscript. LFBP conceived the study, participated in its design and drafted the manuscript. All authors read and approved the final manuscript.

## Authors’ information

PAVS received his M.Sc. in Renewable Energy working on this project. TTT is a laboratory technician at CICY working in the Renewable Energy Department. BBCC is head of the Biotechnology Department at CICY. Her research interests are on enzymatic hydrolysis. ALS is a researcher in the Natural Resources Department at CICY. His main area of expertise is Biomass and Productivity. LFBP is head of the Renewable Energy Department. His research area involves biofuel production.
